# Ibudilast, a Pharmacologic Phosphodiesterase Inhibitor, Prevents Human Immunodeficiency Virus-1 Tat-Mediated Activation of Microglial Cells

**DOI:** 10.1371/journal.pone.0018633

**Published:** 2011-04-08

**Authors:** Michelle Kiebala, Sanjay B. Maggirwar

**Affiliations:** Department of Microbiology and Immunology, University of Rochester School of Medicine and Dentistry, Rochester, New York, United States of America; George Mason University, United States of America

## Abstract

Human Immunodeficiency Virus-1 (HIV-1)-associated neurocognitive disorders (HAND) occur, in part, due to the inflammatory response to viral proteins, such as the HIV-1 transactivator of transcription (Tat), in the central nervous system (CNS). Given the need for novel adjunctive therapies for HAND, we hypothesized that ibudilast would inhibit Tat-induced excess production of pro-inflammatory cytokines, such as tumor necrosis factor-alpha (TNFα) in microglial cells. Ibudilast is a non-selective cyclic AMP phosphodiesterase inhibitor that has recently shown promise as a treatment for neuropathic pain via its ability to attenuate glial cell activation. Accordingly, here we demonstrate that pre-treatment of both human and mouse microglial cells with increasing doses of ibudilast inhibited Tat-induced synthesis of TNFα by microglial cells in a manner dependent on serine/threonine protein phosphatase activity. Ibudilast had no effect on Tat-induced p38 MAP kinase activation, and blockade of adenosine A_2A_ receptor activation did not reverse ibudilast's inhibition of Tat-induced TNFα production. Interestingly, ibudilast reduced Tat-mediated transcription of TNFα, via modulation of nuclear factor-kappa B (NF-κB) signaling, as shown by transcriptional activity of NF-κB and analysis of inhibitor of kappa B alpha (IκBα) stability. Together, our findings shed light on the mechanism of ibudilast's inhibition of Tat-induced TNFα production in microglial cells and may implicate ibudilast as a potential novel adjunctive therapy for the management of HAND.

## Introduction

Human Immunodeficiency Virus-1 (HIV-1) enters the central nervous system (CNS) early after infection, and in many cases may result in a variety of neurological defects collectively termed HIV-1 associated neurocognitive disorders (HAND) [1]. HAND can include neurocognitive impairments, motor deficits, or dementias [2], and continues to significantly impair quality of life despite efficacious reduction of viral load by highly active anti-retroviral therapy (HAART) [3], [4], [5]. Traditionally, the onset of HAND correlated with CNS viral load, and the neuropathological features included multinucleated giant cells, reactive astrocytosis, myelin pallor, and neuronal loss [6], [7], [8], [9]. Recent neuropathologic reports of severe white matter damage (i.e. leukoencephalopathy) in patients with HIV-1 infection and on HAART with very low viral load [10], [11], [12], [13], suggest that additional patterns of primary brain disease are emerging, possibly due to as yet unexplained interactions between the virus, vulnerable populations of neural cells, and HAART [14], [15].

The pathogenesis of HAND likely involves a toxic combination of secreted factors released from HIV-1 infected, brain-resident macrophage and glia, and oxidative stress, which together impair neuronal function. HIV-1 productively infects microglia and perivascular macrophage, the resident phagocytes of the CNS, but does not infect neurons. This suggests that HIV-1 indirectly contributes to the neuropathology seen in HAND patients. Accordingly, neurologic deficits in HAND are more closely correlated with the presence of activated macrophage and microglia than with the amount of neuronal apoptosis or viral RNA [16], [17], [18]. Soluble viral proteins such as Tat and the glycoprotein gp120 can be released from infected microglia and macrophage [19]. Circulating Tat levels have been measured in patient sera from HIV-1 positive individuals, at levels ranging from 1–40 ng/mL [20], [21], however, local extracellular concentrations in the CNS may be much higher, particularly in close proximity to HIV-1 positive perivascular cells [22]. Tat can also interact with and activate neighboring, uninfected cells including microglia, astrocytes, and neurons. Both infected and activated microglia and astrocytes produce pro-inflammatory cytokines including tumor necrosis factor-alpha (TNFα) and interleukin-1 beta (IL-1β), which further activate neighboring cells. Infected and activated cells also produce chemokines such as monocyte chemotactic protein-1 (MCP-1), thereby attracting more inflammatory monocytes and macrophage [23], [24]. Thus, circulating Tat is very likely involved in triggering this vicious inflammatory cycle, eventually leading to neuron damage and cognitive deficits [20].

It is clear that despite effective control of systemic HIV-1 levels with HAART, cognitive impairment still persists with a high prevalence. Considering the failure of antiretroviral therapies to prevent or reverse cognitive decline mediated by HIV-1, recent focus has shifted to the development of adjunctive therapies that specifically target neurocognitive impairment. General classes of drugs being explored in clinical trials include anti-inflammatory agents such as minocycline (NCT00361257 - http://www.clinicaltrials.gov identifier), antioxidants such as selegiline [25], [26], and anti-excitotoxic drugs such as memantine [27]. Only memantine, which is an N-methyl-D-aspartic acid receptor (NMDAR) blocker, has shown potential neuroprotective properties as determined by magnetic resonance spectroscopy [27]. Given the need for novel adjunctive therapies for HAND, we hypothesized that the phosphodiesterase inhibitor, ibudilast, would inhibit Tat-induced, excess production of pro-inflammatory cytokines, such as TNFα, in microglial cells.

Ibudilast is a relatively non-selective cyclic AMP phosphodiesterase (PDE) inhibitor that has been used for decades in Japan to treat bronchial asthma and post-stroke dizziness [28], [29]. Ibudilast has also recently shown promise as a treatment for neuropathic pain, in multiple rat models, via its ability to attenuate glial cell activation [30]. Interestingly, *in vitro* experiments have shown that ibudilast has potential as an anti-inflammatory agent, as it can inhibit lipopolysaccharide (LPS)-induced cytokine production in microglial cells [31], [32]. Ibudilast is also currently being tested in clinical trials as a treatment for multiple sclerosis (MS), opioid withdrawal, and neuropathic pain, all of which are conditions involving aberrant microglial activation and CNS inflammation [28], [33], [34]. Other PDE inhibitors, pentoxifylline and rolipram, have been investigated as anti-inflammatory agents, and have been shown to inhibit HIV-1 replication [35], [36], [37]. Because of its approval for use in humans, as well as its ability to cross the blood brain barrier and inhibit glial cell activation, ibudilast is an exciting potential adjunctive therapy for HAND [30]. Here we investigate the anti-inflammatory properties of ibudilast in the context of HIV-1-induced neuroinflammation.

Initial experiments in human microglial cells demonstrated that ibudilast did indeed inhibit Tat-induced pro-inflammatory cytokine production in these cells. Similarly, in BV-2 mouse microglial cells, ibudilast had an inhibitory effect on Tat-induced TNFα production. These results are in agreement with previous reports of ibudilast's ability to attenuate glial cell activation. Further *in vitro* experiments were performed to determine the mechanisms of ibudilast's inhibition of TNFα release from microglial cells. These experiments suggested that ibudilast inhibits Tat-mediated transcription of TNFα via modulation of nuclear factor-kappa B (NF-κB) signaling. Together, our findings shed light on the mechanism of ibudilast's inhibition of Tat-induced TNFα production in microglial cells and may implicate ibudilast as a potential novel adjunctive therapy for the management of HAND.

## Methods

### Reagents

HIV-1 Tat 1–72 was obtained from Philip Ray (University of Kentucky, Lexington, KY, USA). This recombinant Tat protein was used at 100 nM (∼800 ng/mL) concentration, unless otherwise noted. Soluble Tat levels in HIV-1 patient sera have been measured up to 40 ng/mL [20], [21]. It has also been shown that Tat can interact with endogenous glycosaminoglycans and heparan sulfates, thereby potentially lowering its measurable concentration *in vivo* [38]. Therefore, it is likely that Tat concentrations surrounding HIV-1-infected cells are much higher [22]. In addition, Tat's exceedingly strong affinity for other proteins and glass/plastic, and its temperature susceptibility, make it impossible to determine exactly what fraction of the Tat “starting dose” actually reaches the experimental specimen, thus likely leading to an underestimation of Tat functions *in vitro* [39]. Finally, Tat's effects *in vivo* are likely to occur over long-term chronic exposures. Chronic, low dose *in vivo* effects of any reagent are often appropriately modeled *in vitro*, by proportionately higher doses of that same reagent, over more acute time frames. For these reasons, and in order to be consistent with previous experiments from our laboratories, and others, we use 100 nM Tat for these experiments, which is at the lower end of the Tat dose range seen with many comparable studies, and which many studies have found to be an appropriate Tat dose by which to model Tat's *in vivo* effects *in vitro*.

Ibudilast was obtained from Santa Cruz Biotechnologies Inc., (Santa Cruz, CA, USA). Okadaic acid (Oka) was purchased from Calbiochem/Millipore (Billerica, MA, USA). SB203580 and 3-[4,5-Dimethylthiazol-2-yl]-2,5-diphenyltetrazolium bromide (MTT) were purchased from Sigma-Aldrich (St. Louis, MO, USA). ZM241385 and CGS21680 were purchased from Tocris Bioscience, (Ellisville, MO, USA). MG-132 was purchased from BioMol International/Enzo Life Sciences (Plymouth Meeting, PA, USA). DMSO was used as a vehicle control. Anti-Tat antibody was obtained from the AIDS Research and Reference Reagent Program (Germantown, MD, USA) and non-immune control serum (IgG) was obtained from Santa Cruz Biotechnologies Inc., (Santa Cruz, CA, USA). Both antibodies were used at a concentration of 8 µg/mL.

### Cell Cultures

Human microglial cells that were isolated from fetal human brain were obtained from Clonexpress (Gaithersburg, MD, USA) and were maintained in 50∶50 DMEM: F-12 supplemented with 5% FBS and 10 ng/mL of M-CSF.

The murine microglial cell-line (BV-2) was obtained from Dr. R. Donato (University of Perugia, Perugia, Italy). These cells were maintained in DMEM containing 10% FBS, 2 mM glutamine, and antibiotics, by standard procedures.

### ELISA

TNFα levels were measured in culture supernatants (pre-cleared by brief centrifugation) by using a mouse TNFα ELISA kit (eBioscience, San Diego, CA, USA) according to the manufacturer's instructions. This kit has a minimum sensitivity threshold of 8 pg/mL. Briefly, 50 µL of cell culture supernatant was incubated in a 96-well plate pre-coated with a TNFα-specific monoclonal antibody for 1.5 h. After extensive washing, binding of TNFα was detected by incubation with biotinylated antibodies, followed by streptavidin-peroxidase; colorimetric enzyme assays were performed to detect bound TNFα.

Other cytokine levels were measured using Bio-Plex Multi-Plex analysis for detecting a panel of multiple cytokines from a single sample (Bio-Rad, Hercules, CA, USA). Briefly, this technology uses multiple spectrally identifiable polystyrene beads, each coated with a different anti-cytokine antibody, followed by target, then secondary antibody binding, to detect multiple cytokines from a single sample in typical sandwich-assay fashion. This assay was utilized to measure levels of TNFα, IL-1β, IL-6, and MCP-1 in Tat-treated, primary human microglia supernatant samples, as previously described [40], [41].

### MTT Assay

BV-2 cells (1.2×10^5^) were plated in a 24-well plate and were treated with increasing concentrations of ibudilast or DMSO control for 7 h. 10× MTT was added to the wells and the cells were incubated for an additional 1 h at 37°C. After removal of the media, MTT solvent (4 mM HCl, 0.1% NP40, in isopropanol) was added to dissolve the formazan dye. The optical density was measured at 570 nm and the percent cell survival was determined compared to the untreated (NT) sample.

### p38 MAP Kinase Activity Assay

p38 MAP kinase activity levels were measured in BV-2 cell lysates using a p38 kinase activity kit (Cell Signaling Technology, Danvers, MA, USA) according to the manufacturer's instructions. Briefly, phosphorylated p38 was immunoprecipitated from 200 µL of whole cell lysate using a phospho-p38 MAP kinase monoclonal antibody crosslinked to agarose hydrazide beads. The antibody-bead pellet was then incubated with an Activating Transcription Factor-2 (ATF-2) fusion protein as the substrate. ATF-2 phosphorylation was detected by immunoblot analysis using a phospho-ATF-2 (Thr71) antibody. Equal protein content among the samples was verified using immunoblot analysis for total p38 and α-Tubulin levels in the cell lysate supernatant that was collected after the immunoprecipitation.

### Plasmids

The luciferase reporter construct driven by NF-κB and the RelA expression plasmid were obtained from Dr. S. C. Sun (MD Anderson Cancer Center, University of Texas, Houston, TX, USA). The luciferase reporter construct driven by the mouse TNFα (mTNFα) promoter was obtained from Dr. Dmitry Kuprash (Engelhardt Institute of Molecular Biology, Moscow, Russia).

### Transient Transfections

BV-2 cells were transfected with plasmid DNA using Nucleofector (Amaxa/Lonza, Basel, Switzerland; Walkersville, MD, USA). For nucleofection, 10×10^6^ cells were transfected with 10 µg total plasmid DNA. Transfected cells were plated at 3.5×10^5^ cells/well in a 24-well plate and incubated for 24 h prior to treatment. Media was changed 4 h after transfection and again prior to treatment to reduce cell toxicity. In order to determine transfection efficiency, a separate aliquot of BV-2 cells was transfected with a p-Max-GFP expressing vector (Amaxa/Lonza, Basel, Switzerland; Walkersville, MD, USA), and 24 h post-transfection, greater than 90% of BV-2 cells were GFP-positive.

### Luciferase Assays

Luciferase reporter plasmids containing either NF-κB responsive elements upstream of a firefly luciferase gene or the mouse TNFα promoter region upstream of a firefly luciferase gene were transfected into BV-2 cells using Nucleofector (Amaxa/Lonza, Basel, Switzerland; Walkersville, MD, USA). 24 h post-transfection, cells were either left untreated or were incubated for 8 h with 100 nM Tat. Cell lysates were prepared using 1× reporter lysis buffer (Promega Life Sciences, Madison, WI, USA), and luciferase activity was measured with a SpectraMax M3 Plate Reader (Molecular Devices, Sunnyvale, CA). In these assays, total protein amount as determined by Bradford assay (Bio-Rad, Hercules, CA, USA), was used to normalize the samples. In the case of luciferase assays without Tat treatment, co-transfection with a *Renilla* luciferase reporter driven by the constitutively active herpes simplex virus type 1 thymidine kinase (HSV-1 TK) promoter was used as an internal control for transfection efficiency. In the case of luciferase assays with Tat treatment, parallel transfection with a luciferase reporter plasmid containing responsive elements of the OCT-1 transcription factor upstream of a firefly luciferase gene was used as a control for the specificity of Tat's effect on transcription.

### Protein Phosphatase Activity Assay

BV-2 cells (4.6×10^5^) were plated in 6-well plates and were treated with 50 µM ibudilast for the indicated periods of time. Cells were washed in phosphate buffered saline (PBS) and whole cell lysates were collected in ELB buffer (50 mM HEPES (pH 7), 250 mM NaCl, 0.1% Nonidet P-40, 5 mM EDTA, 10 mM NaF, 0.1 mM Na_3_VO_4_, 50 µM ZnCl_2_, supplemented with 0.1 mM PMSF, 1 mM DTT, and a mixture of protease inhibitors, phosphatase inhibitors were not added). PP2A activity was measured with a Ser/Thr phosphatase assay kit (Promega Life Sciences, Madison, WI, USA) according to the manufacturer's protocol. Briefly, samples containing 5 µg of cell protein were added to a solution containing the peptide substrate (100 µM) in a 96-well plate and incubated for 10 min. Then a Malachite Green solution was added (50 µL) and absorbance (560 nm) was measured on a DTX 880 Microplate Reader (Beckman Coulter, Brea, CA). Phosphate release was determined by comparing absorbance with a standard curve.

### Immunoblotting Assays

Following the indicated treatments, whole cell lysates were prepared in ELB buffer (50 mM HEPES (pH 7), 250 mM NaCl, 0.1% Nonidet P-40, 5 mM EDTA, 10 mM NaF, 0.1 mM Na_3_VO_4_, 50 µM ZnCl_2_, supplemented with 0.1 mM PMSF, 1 mM DTT, and a mixture of protease and phosphatase inhibitors). Cellular debris was removed by high-speed centrifugation. Lysates were fractionated on 7.5% SDS-PAGE gels and protein was electrophoretically transferred to Hybond ECL nitrocellulose membrane (GE Healthcare Bio-Sciences Corporation, Piscataway, NJ, USA). The membranes were analyzed for immunoreactivity with primary antibodies raised against IκBα (1∶1000), RelA (1∶1000), or α-Tubulin (1∶1000; all from Santa Cruz Biotechnologies Inc., Santa Cruz, CA, USA), p38 MAP kinase (1∶1000), Phospho-p38 MAPK (1∶1000), RelA P-S276 (1∶1000), RelA P-S536 (1∶1000), or PP2Ac (1∶1000; all from Cell Signaling Technology, Danvers, MA, USA). Bound antibodies were detected by species-specific, horseradish peroxidase (HRP)-conjugated secondary antibodies (1∶3000, GE Healthcare Bio-Sciences Corporation, Piscataway, NJ, USA), followed by addition of ECL reagent (Pierce Biotechnology/Thermo Fisher Scientific, Rockford, IL, USA) and subsequent exposure to x-ray film. Equal loading and uniformity of protein transfer to the nitrocellulose membrane were verified by stripping and reprobing the membranes with primary antibodies specific to α-Tubulin.

### Real-Time RT-PCR Analyses

Total RNA was isolated from treated BV-2 cells (2.5×10^5^) using the Roche High Pure RNA Isolation kit (Roche Applied Science, Indianapolis, IN, USA) according to the manufacturer's protocol. Complementary DNA (cDNA) synthesis was performed using 2 µg total RNA, oligo-dT primers, and the Superscript III first-strand synthesis system (Invitrogen, Carlsbad, CA, USA). Gene-specific primer sequences were as follows: 1) TNFα primers: forward 5′-ACTCCAGAACATCTTGGAAATAGC-3′, reverse 5′-GCGGATCATGCTTTCTGTGC-3′; 2) GAPDH primers: forward 5′-TGATGACATCAAGAAGGTGGTGAA-3′, reverse 5′-TCCTTGGAGGCCATGTAGGCCAT-3′. Real-time RT-RCR was performed using iQ™ SYBR® Green PCR Supermix (Bio-Rad, Hercules, CA, USA) and 100 nM gene-specific forward and reverse primers in 20 µL total volume. After denaturation for 3 min. at 95°C, the PCR was run for 40 cycles of 95°C for 30 sec., primer-specific melting (T_m_ °C) for 1 min., and 72°C for 30 sec. using an iCycler instrument (Bio-Rad, Hercules, CA, USA). GAPDH served as an internal control in these experiments.

### Statistical Analysis

Mean data values and the standard error of the mean (SEM) were calculated for each variable. One-way ANOVA followed by Bonferroni's test for multiple comparisons was used to analyze data involving the analysis of multiple sample groups. A value of p<0.05 was designated as statistically significant.

## Results

### Ibudilast inhibits Tat-induced pro-inflammatory cytokine production in microglial cells

**Figure 1:**
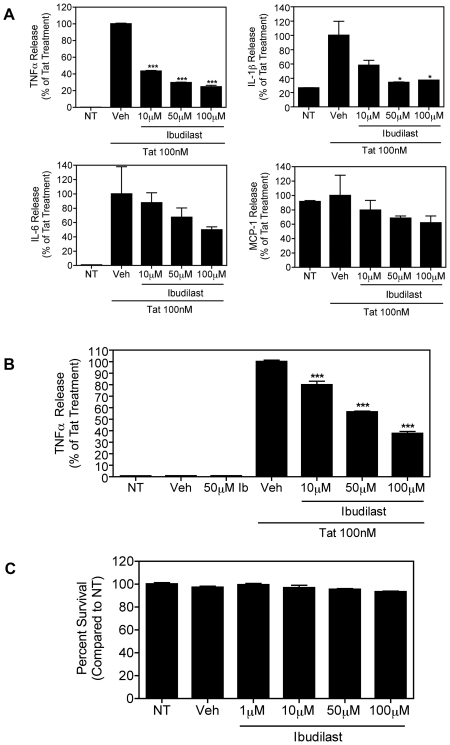
Ibudilast dose-dependently inhibits Tat-induced pro-inflammatory cytokine production in microglial cells. *A*, Human microglial cells (1×10^5^) were left untreated (NT) or were treated with Tat (100 nM) for 8 h with or without pre-treatment for 30 min. with increasing concentrations of ibudilast or vehicle (Veh), as indicated. TNFα, IL-1β, IL-6, and MCP-1 levels in culture supernatants were analyzed by Multi-Plex cytokine array, as described in Methods. The Tat+Veh-treated samples were set to 100% and all other samples were compared to this value. Results are shown as mean ± SD of values derived from two replicates from a single representative experiment. Statistical significance (***, p<0.001; *, p<0.05) is indicated, as compared to Tat+Veh-treated cells. The average cytokine/chemokine concentration of the Tat+Veh-treated sample was as follows; TNFα: 8166 pg/mL, IL-1β: 3.3 pg/mL, IL-6: 6027 pg/mL, and MCP-1: 1480 pg/mL. *B*, Similarly, murine microglial cells (BV-2; 1.2×10^5^) were left untreated (NT) or were treated with Tat (100 nM) for 8 h with or without pre-treatment for 30 min. with increasing concentrations of ibudilast or vehicle (Veh), as indicated. TNFα release was measured by ELISA. Results are shown as mean ± SEM of values derived from three replicates from a single representative experiment; three total experiments were performed. Statistical significance (p<0.001) is indicated, as compared to Tat+Veh-treated cells (***). The TNFα concentration of the Tat+Veh-treated sample was 1861 pg/mL. *C*, BV-2 cells (1.2×10^5^) were left untreated (NT) or were treated with increasing concentrations of ibudilast or vehicle (Veh), as indicated, for 8 h. The MTT assay was used as a measure of cell viability. Percent survival was calculated as compared to the untreated sample. Results are shown as mean ± SEM of values derived from four replicates from a single representative experiment; two total experiments were performed.

Multiple reports have shown that ibudilast can inhibit both LPS and interferon-gamma (IFN-γ)-induced production of pro-inflammatory molecules in microglial cells, thus highlighting the anti-inflammatory activities of this drug [32], [42]. This led us to hypothesize that ibudilast may inhibit Tat's effects on microglial cells. Although exposure of microglial cells to LPS, IFN-γ, and Tat can all similarly result in cytokine synthesis and NF-κB activation, the upstream signaling events leading to this cytokine production are vastly different among these varying stimuli. For example, LPS is recognized by Toll-like receptor 4 (TLR4), leading to NF-κB activation, whereas IFN-γ activates the IFN-γ receptor (IFN-γR), leading to activation of the Janus kinase (JAK)/signal transducers and activators of transcription (STAT) pathway. Alternatively, exposure of microglial cells to Tat does not result in TLR or IFN-γR activation. Extracellular Tat can enter cells in an active form [38], [43], leading to the activation of multiple signaling pathways including NF-κB and MAP kinase cascades. Activated microglia are believed to be centrally involved in HAND-associated neuropathology as pro-inflammatory factors, such as TNFα, released from infected and activated macrophage and glial cells play a central role in the inflammatory cycle that eventually leads to neuron damage and cognitive deficits [44], [45]. We first used a multi-plex cytokine array assay to determine whether ibudilast altered Tat-induced pro-inflammatory cytokine and chemokine production in human microglial cells. These results (Figure 1A) indicated a profound reduction in Tat-induced production of TNFα and IL-1β by ibudliast. Although not significant, there was also a trend toward dose-dependent inhibition of Tat-mediated IL-6 and MCP-1 production by ibudilast. Our results for MCP-1 are in agreement with previous reports that human monocytes and monocyte-derived macrophage have high basal levels of MCP-1 production in the absence of pro-inflammatory stimuli [46], [47]. Based on previously published results, MCP-1 production is induced after 12–24 h incubation with Tat [48], [49]. Therefore, it is likely that with a longer treatment time with Tat, we would have seen a more robust increase in MCP-1 production. In addition, little is known about the temporal relationship between TNFα, IL-1β, IL-6, and MCP-1 production caused by Tat in microglial cells *in vivo*. These results in human microglial cells strongly support an anti-inflammatory role of ibudilast.

We next focused on ibudilast's ability to inhibit Tat-induced TNFα synthesis in the mouse microglial BV-2 cell line, which is genetically manipulatable. These cells are also widely used for studies related to inflammation induced by generic inflammatory mediators [50], [51], [52], and those encoded by HIV-1 [53], [54]. Following an 8 h 100 nM Tat treatment of these cells, we collected culture supernatants and measured TNFα levels by ELISA. The results shown in Figure 1B reveal the ability of ibudilast to inhibit Tat-mediated TNFα production in mouse microglial BV-2 cells, further supporting an anti-inflammatory role for ibudilast in the context of HIV-1. As shown in Figure 1, there is slightly less inhibition of TNFα by ibudilast in BV-2 cells than in human microglial cells. This could be due to the fact that BV-2 cells are rapidly dividing, whereas the human microglial cells are non-dividing. We also confirmed that ibudilast's inhibition of TNFα production was not due to cellular toxicity. The MTT assay was used as a measure of cell viability. As shown in Figure 1C, treatment of BV-2 cells with increasing concentrations of ibudilast did not result in significant cell death.

### Blockade of adenosine A_2A_ receptor does not reverse ibudilast's inhibition of Tat-mediated TNFα production

**Figure 2:**
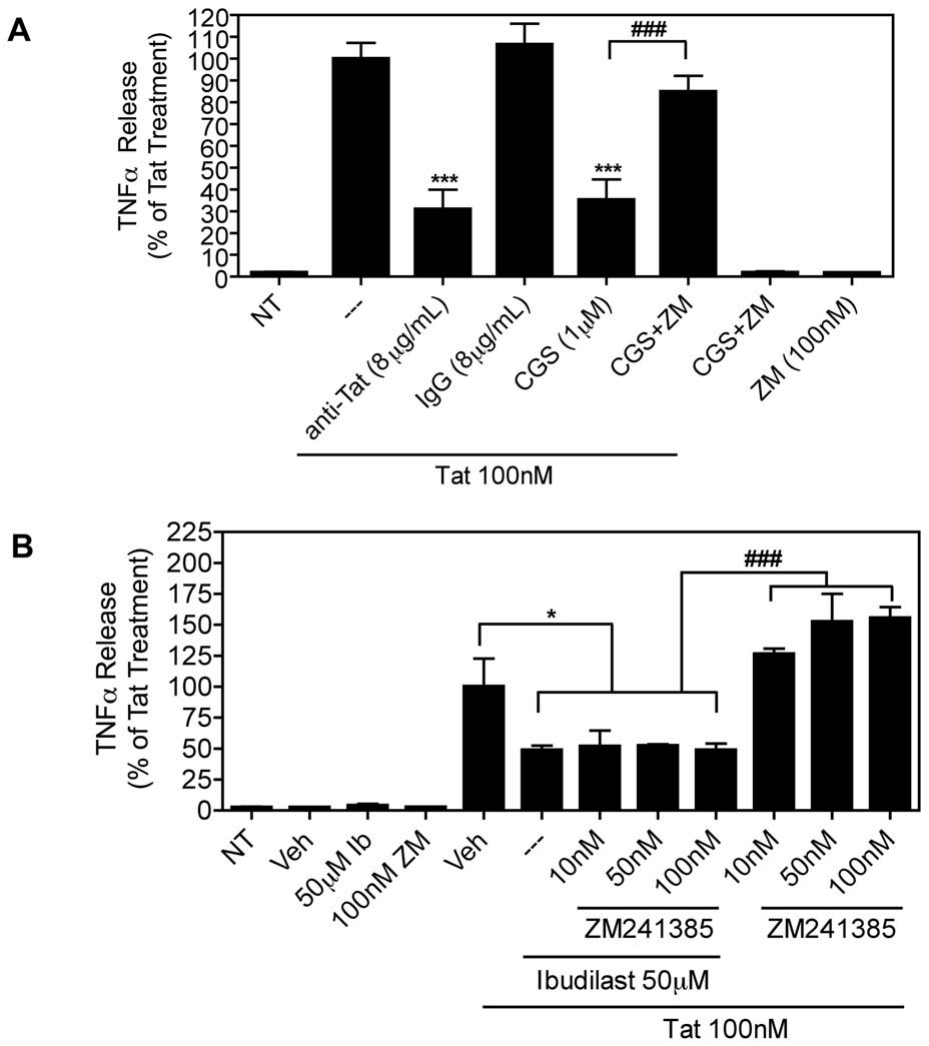
Inhibition of Tat-induced TNFα production by ibudilast is not reversed by blockade of adenosine A_2A_ receptor. *A*, BV-2 cells (1.5×10^5^) were left untreated (NT) or were treated with Tat (100 nM) for 8 h with or without pre-treatment for 30 min. with either anti-Tat or control IgG antibodies (8 µg/mL) or adenosine A_2A_ receptor agonist, CGS21680 (CGS, 1 µM) or antagonist, ZM241385 (ZM, 100 nM), as indicated. TNFα release was measured by ELISA. The Tat-treated samples were set to 100% and all other samples were compared to this value (the TNFα concentration for this sample was 3475 pg/µg total protein content). Results are shown as mean ± SEM of values derived from four replicates from a single representative experiment; two total experiments were performed. Statistical significance (p<0.001) is indicated, as compared to Tat-treated cells (***) or as compared to Tat+CGS-treated cells (###). *B*, BV-2 cells (1.2×10^5^) were left untreated (NT) or were treated with Tat (100 nM) for 8 h with or without pre-treatment for 30 min. with ibudilast (Ib, 50 µM) or vehicle (Veh) alone or together with increasing concentrations of the adenosine A_2A_ receptor antagonist, ZM241385, as indicated. TNFα release was measured by ELISA. The Tat+Veh-treated samples were set to 100% and all other samples were compared to this value (the TNFα concentration for this sample was 696.2 pg/mL). Results are shown as mean ± SEM of values derived from three replicates from a single representative experiment; two total experiments were performed. Statistical significance is indicated, as compared to Tat+Veh-treated cells (p<0.05, *) or as compared to Tat+ZM241385-treated cells (p<0.001, ###).

Next we examined the mechanisms by which ibudilast inhibits Tat-induced TNFα production. PDE inhibition is known to affect adenosine receptor activation [55], [56], [57], and adenosine is an important director of inflammatory responses [58], [59], [60]. Adenosine receptor activation can inhibit the production of TNFα, and in the context of HIV-1, it has been shown that adenosine A_2A_ receptor activation inhibits Tat-induced TNFα production in human monocytes [61]. Therefore, we speculated that ibudilast's inhibition of Tat-induced TNFα production might involve modulation of adenosine A_2A_ receptor activation. As shown in Figure 2A, we confirmed that activation of the A_2A_ receptor with the agonist CGS21680 (1 µM) inhibited Tat-induced TNFα production in mouse microglial cells as measured by TNFα ELISA. This inhibition was reversed with the A_2A_ receptor antagonist ZM241385 (100 nM). Pre-treatment with an anti-Tat antibody also blocked Tat-induced TNFα production, whereas a control non-immune IgG had no effect on Tat-induced TNFα. This indicated that the TNFα production was Tat-specific (Figure 2A). However, blockade of A_2A_ receptor activation with increasing concentrations of ZM241385 did not reverse the inhibition of Tat-induced TNFα release by ibudilast in mouse microglial cells (Figure 2B), suggesting that ibudilast's inhibition of TNFα does not involve adenosine A_2A_ receptor activation.

### Tat-induced activation of p38 MAP kinase is not affected by ibudilast

**Figure 3:**
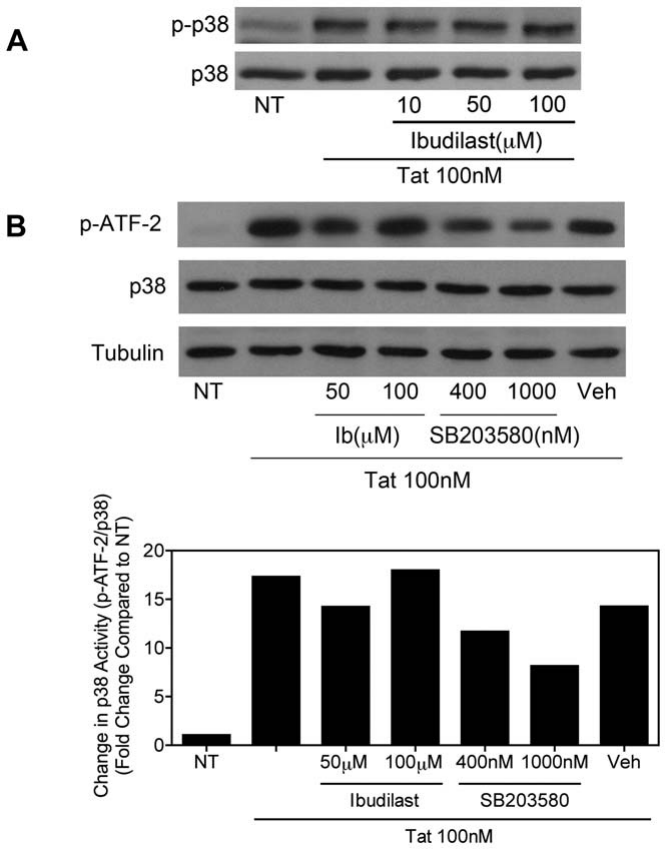
Ibudilast does not inhibit Tat-induced activation of p38 MAP kinase. *A*, BV-2 cells (1.2×10^5^) were left untreated (NT) or were treated with Tat (100 nM) for 15 min. with or without pre-treatment for 5 min. with increasing concentrations of ibudilast, as indicated. Whole cell lysates were subjected to immunoblot analysis using either phospho-p38 MAPK-specific *(upper panel)* or p38 MAPK-specific *(lower panel)* antibodies. The results of a single representative experiment are shown; three total experiments were performed. *B*, BV-2 cells (2.4×10^5^) were left untreated (NT) or were treated with Tat (100 nM) for 20 min. with or without pre-treatment for 10 min. with increasing concentrations of ibudilast or the p38 MAP kinase inhibitor, SB203580, as indicated. Whole cell lysates were subjected to immunoprecipitation with an immobilized phospho-p38 MAP kinase antibody. Precipitated p38 was incubated with an ATF-2 fusion protein substrate. Phosphorylated ATF-2 levels were determined by immunoblot analysis with a phospho-ATF-2-specific antibody (*top panel*). Whole cell lysate supernatants collected after immunoprecipitation were subjected to immunoblot analysis using p38-specific (*middle panel*) or α-Tubulin-specific (*lower panel*) antibodies. Protein levels were quantified using ImageJ software *(bottom graph)*. Phospho-ATF-2 levels were normalized to total p38 MAPK levels and fold change compared to NT was calculated. The results of a single representative experiment are shown; two total experiments were performed.

To further examine the mechanisms of ibudilast's inhibition of Tat-mediated TNFα production, we looked at the activation of p38 MAP kinase, which is known to play an important role in the production of pro-inflammatory cytokines [62]. p38 MAP kinase is also a known target for other PDE inhibitors such as pentoxifylline [63]. Using an antibody that recognizes phosphorylated p38, we did see an increase in the phosphorylation of p38 induced by 15 min. 100 nM Tat treatment (Figure 3A). Pre-treatment with increasing concentrations of ibudilast did not affect Tat-induced p38 phosphorylation. Under these conditions total p38 levels (protein loading control) were not altered. In addition, there was an approximately 15-fold increase in p38 MAP kinase activity induced by 20 min. 100 nM Tat treatment, as measured by an *in vitro* kinase assay (Figure 3B). Pre-treatment with ibudilast did not inhibit this Tat-induced increase in p38 activity. As a positive control, the p38 inhibitor, SB203580, did reduce Tat-mediated p38 kinase activity. Densitometric analysis of the phospho-ATF-2 bands versus the total p38 bands is shown in Figure 3B.

### Ibudilast inhibits Tat-induced TNFα production through serine/threonine protein phosphatase activity

**Figure 4:**
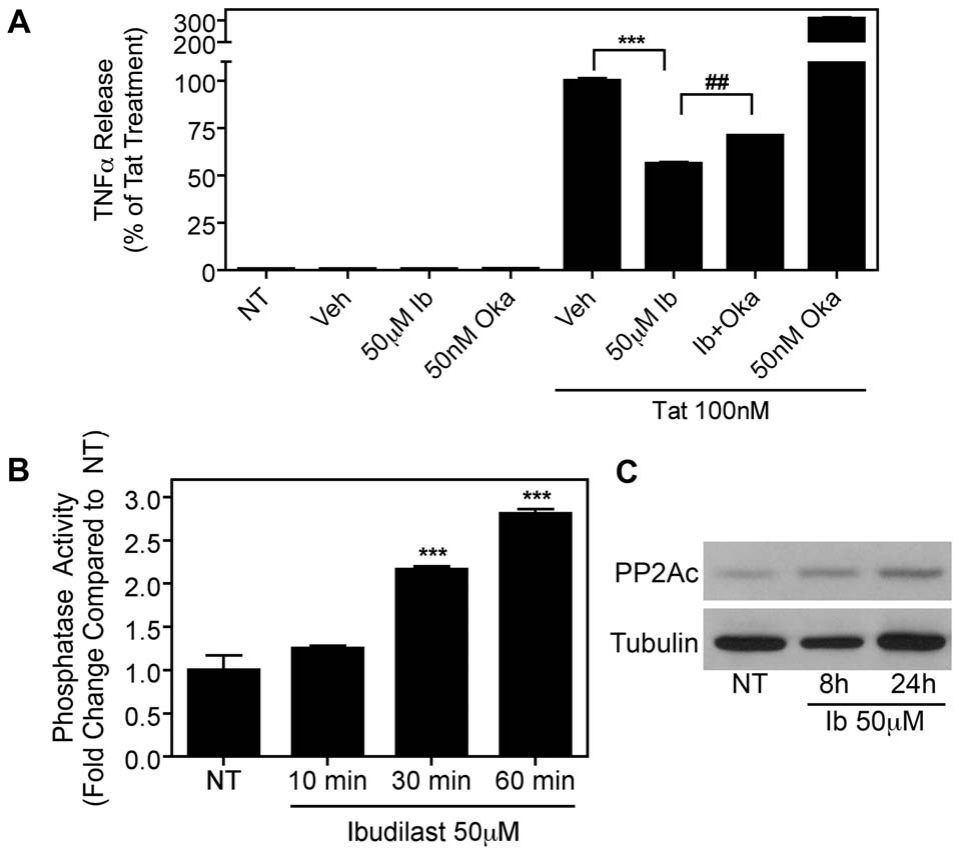
Ibudilast inhibits Tat-induced TNFα production in a protein phosphatase dependent manner. *A*, BV-2 cells (1.2×10^5^) were left untreated (NT) or were treated with Tat (100 nM) for 8 h with or without pre-treatment for 30 min. with ibudilast (Ib, 50 µM) or vehicle (Veh) alone or together with the protein phosphatase inhibitor, okadaic acid (Oka, 50 nM). TNFα release was measured by ELISA. The Tat+Veh-treated samples were set to 100% and all other samples were compared to this value (the TNFα concentration for this sample was 1861 pg/mL). Results are shown as mean ± SEM of values derived from three replicates from a single representative experiment; two total experiments were performed. Statistical significance is indicated, as compared to Tat+Veh-treated cells (p<0.001, ***) or as compared to Tat+Ib-treated cells (p<0.01, ##). *B*, Serine/Threonine phosphatase activity was measured in whole cell lysates from BV-2 cells (4.6×10^5^) treated with ibudilast (50 µM) for the indicated periods of time. Results are shown as mean ± SEM of values derived from three replicates from a single representative experiment; two total experiments were performed. Statistical significance (p<0.001) is indicated, ***. *C*, BV-2 cells (1.2×10^5^) were left untreated (NT) or were treated with ibudilast (50 µM) for 8 h or 24 h, as indicated. Whole cell lysates were subjected to immunoblot analysis using either PP2Ac-specific *(upper panel)* or α-Tubulin-specific *(lower panel)* antibodies. The results of a single representative experiment are shown; two total experiments were performed.

PDE inhibitors have been shown to modulate protein phosphatase activity [64], [65], and the involvement of protein phosphatases in the inhibition of Tat-induced TNFα production has been well characterized [61]. Thus, we predicted that the inhibitory actions of ibudilast on TNFα production could be regulated by protein phosphatase activation. As expected, ibudilast's inhibition of Tat-induced TNFα production was significantly reversed by okadaic acid (50 nM), which is a serine/threonine protein phosphatase inhibitor (Figure 4A). This indicated that the anti-TNFα effects of ibudilast were protein phosphatase dependent. Additionally, we measured phosphatase activity in whole cell extracts from ibudilast-treated BV-2 cells. Ibudilast significantly increased serine/threonine phosphatase activity after 60 min. (Figure 4B). Besides increasing phosphatase activity, ibudilast treatment also increased levels of the catalytically active PP2Ac subunit as detected by immunoblot analysis (Figure 4C). Together these results indicate that ibudilast treatment leads to higher PP2A activity.

### Ibudilast's reduction of Tat-mediated TNFα is at the transcriptional level

**Figure 5:**
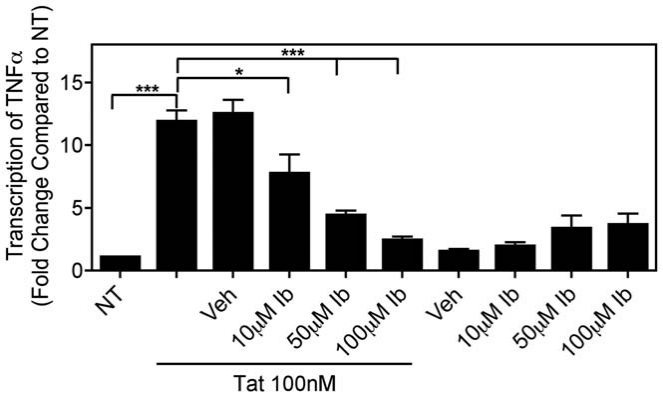
Ibudilast inhibits Tat-induced TNFα transcript levels. BV-2 cells (2.5×10^5^) were left untreated (NT) or were treated with Tat (100 nM) for 4 h with or without pre-treatment for 30 min. with increasing concentrations of ibudilast (Ib) or vehicle (Veh). Total RNA was collected, reverse transcribed using oligo-dT primers, and subjected to Real-Time SYBR Green RT-PCR amplification. Fold induction of TNFα mRNA species was normalized to those of GAPDH and presented as a function of the expression level in NT samples. Data represent mean ± SEM of values derived from three replicates from a single representative experiment. Statistical significance (***, p<0.001 or *, p<0.05) is denoted as compared to Tat-treated samples.

Considering that protein phosphatase activation can affect multiple signaling pathways [66], [67], [68], we predicted that ibudilast might be inhibiting TNFα production at the transcriptional level. We used quantitative real-time PCR (qRT-PCR) to determine whether ibudilast altered Tat-induced TNFα transcript levels in mouse microglial cells. Total RNA isolated from BV-2 cells treated with Tat with or without ibudilast pre-treatment was used to measure the abundance of TNFα transcripts. As compared to non-treated cells, there was an approximately 12-fold increase in TNFα mRNA levels in Tat-treated cells. Pre-treatment with vehicle control did not reduce the Tat-mediated increase in TNFα mRNA levels, however, pre-treatment with increasing concentrations of ibudilast dose-dependently inhibited Tat-induced TNFα mRNA levels (Figure 5).

### Inhibition of Tat-mediated TNFα transcription by ibudilast involves modulation of NF-κB

**Figure 6:**
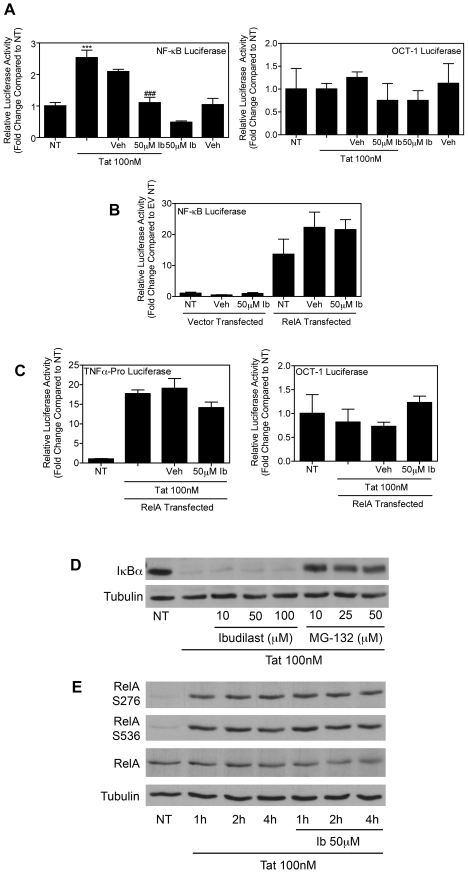
Ibudilast inhibits Tat-induced TNFα production via modulation of NF-κB signaling. *A*, BV-2 cells (10×10^6^) were transiently transfected using Nucleofector (Amaxa/Lonza) with an NF-κB-dependent luciferase reporter plasmid. 16 h post-transfection, cells were left untreated or were treated with Tat (100 nM) for 8 h with or without pre-treatment for 30 min. with 50 µM ibudilast (Ib) or vehicle (Veh). Luciferase activity in whole cell lysates was determined. Results are shown as mean ± SEM of values derived from three replicates from a single representative experiment; two total experiments were performed. Statistical significance is denoted (***, p<0.001) as compared to NT samples or (###, p<0.001) as compared to Tat-treated samples. A parallel transfection with a luciferase reporter containing responsive elements of the OCT-1 transcription factor upstream of a firefly luciferase gene was included as a control for the specificity of ibudilast's inhibition of NF-κB transcriptional activity. *B*, BV-2 cells (10×10^6^) were transiently transfected using Nucleofector (Amaxa/Lonza) with an NF-κB-dependent luciferase reporter plasmid alone or together with either a RelA-encoding plasmid or a vector control plasmid. 16 h post-transfection, cells were left untreated or were treated with 50 µM ibudilast (Ib) or vehicle (Veh) for 8 h. Luciferase activity in whole cell lysates was determined. Results are shown as mean ± SEM of values derived from three replicates from a single representative experiment; two total experiments were performed. Co-transfection with a *Renilla* luciferase reporter plasmid was included as a control for transfection efficiency. For each sample the firefly luciferase reading was divided by the *Renilla* luciferase reading. C, BV-2 cells (10×10^6^) were transiently transfected using Nucleofector (Amaxa/Lonza) with a plasmid containing a luciferase reporter gene under transcriptional control of the mouse TNFα promoter region, alone or together with a RelA-encoding plasmid. 16 h post-transfection, cells were left untreated or were treated with Tat (100 nM) for 8 h with or without pre-treatment for 30 min. with 50 µM ibudilast (Ib) or vehicle (Veh). Luciferase activity in whole cell lysates was determined. Results are shown as mean ± SEM of values derived from three replicates from a single representative experiment; two total experiments were performed. A parallel transfection with the OCT-1 luciferase reporter was included as a control for the specificity of ibudilast's inhibition of TNFα promoter activation. *D*, BV-2 cells (1.2×10^5^) were left untreated (NT) or were treated with Tat (100 nM) for 15 min. with or without pre-treatment for 30 min. with increasing concentrations of ibudilast or MG-132, as indicated. Whole cell lysates were subjected to immunoblot analysis using either IκBα-specific *(upper panel)* or α-Tubulin-specific *(lower panel)* antibodies. The results of a single representative experiment are shown; three total experiments were performed. *E*, BV-2 cells (1.2×10^5^) were left untreated (NT) or were treated with Tat (100 nM) for the indicated periods of time with or without pre-treatment for 30 min. with increasing concentrations of ibudilast, as indicated. Whole cell lysates were subjected to immunoblot analysis using RelA phospho-serine 276-specific *(first panel)*, RelA phospho-serine 536-specific *(second panel)*, total RelA-specific *(third panel)*, or α-Tubulin-specific *(fourth panel)* antibodies. The results of a single representative experiment are shown; two total experiments were performed.

We, and others, have shown that NF-κB signaling plays an important role in Tat-induced TNFα production [54], [69]. Indeed, activation of NF-κB is also affected by protein phosphatase activity [67], [70]. To better understand how ibudilast inhibits Tat-induced TNFα transcription, we used a reporter gene assay. To do this, BV-2 cells were transiently transfected with a luciferase reporter plasmid in which the luciferase gene is placed under direct control of the NF-κB responsive element [71]. These cells were then treated with Tat either alone or together with vehicle control or ibudilast. Our results confirmed that pre-treatment with ibudilast inhibits activation of endogenous NF-κB induced by 8 h 100 nM Tat treatment (Figure 6A). A parallel transfection with a luciferase reporter plasmid in which the luciferase gene is downstream of OCT-1 transcription factor response elements was used as a control for the specificity of ibudilast's inhibition of NF-κB. We performed additional luciferase assays in which BV-2 cells were transfected with the NF-κB reporter together with either a plasmid expressing RelA, which is a major component of active NF-κB dimers, or an empty vector control plasmid. The total amount of DNA in each transfection was kept constant. Over-expression of RelA bypasses signaling defects by enriching functionally active homodimers in the nucleus due to a stoichiometric imbalance between over-expressed RelA and endogenous inhibitors such as p100 and inhibitor of kappa B-alpha (IκBα). Interestingly, we did not see a reduction in luciferase expression induced by over-expression of RelA with ibudilast treatment (Figure 6B). Co-transfection with a *Renilla* luciferase reporter driven by the constitutively active HSV-1 TK promoter was used as an internal control for transfection efficiency. Similarly, BV-2 cells were transfected with the mouse TNFα-promoter luciferase reporter together with a plasmid expressing RelA. These cells were then treated with Tat either alone or together with vehicle control or ibudilast. Our results confirmed that over-expression of RelA was able to overcome ibudilast's inhibition of TNFα promoter activity (Figure 6C). As in Figure 6A, a parallel transfection with an OCT-1 luciferase reporter confirmed the specificity of ibudilast's inhibition of TNFα promoter activation. Together these results suggest that ibudilast inhibits the NF-κB signaling cascade, since this inhibition was overcome by RelA over-expression.

Inhibition of NF-κB can take place at multiple steps within the NF-κB signal transduction pathway. For example, the nuclear translocation of NF-κB proteins such as RelA could be blocked, or the transcriptional activity of these proteins in the nucleus could be inhibited. The IκB molecules interact with NF-κB dimers, leading to their retention in the cytoplasm in resting cells, as well as blocking the DNA binding activity of NF-κB [72]. Upon activation of the NF-κB pathway, IκBα is phosphorylated by the inhibitor of kappa B kinase (IKK) complex and subsequently targeted for ubiquitination and proteolytic degradation, thus freeing NF-κB dimers to move to the nucleus where they can activate transcription [73]. Indeed, high levels of IκBα were detected in whole cell lysates from untreated BV-2 cells, and as expected, incubation of these cells with Tat for 15 min. led to the rapid degradation of IκBα (Figure 6D). However, we did not see a reversal of this Tat-induced IκBα degradation by pre-treatment with increasing concentrations of ibudilast (Figure 6D). Additionally, pre-treatment with a proteasomal inhibitor, MG-132, did significantly reverse the Tat-induced IκBα degradation, as expected [74], [75]. Under these conditions, no change was observed in α-Tubulin protein levels (Figure 6D). Likewise, analogous to the results shown in Figure 6D, using immunofluorescence analysis, we observed nuclear translocation of RelA in 1 h 100 nM Tat-treated BV-2 cells. This RelA nuclear localization was not inhibited by pre-treatment with ibudilast (data not shown).

The transcriptional activity of RelA is partially controlled by post-translational modifications, including phosphorylation [76]. Through the activation of multiple signaling cascades, Tat and Tat-induced factors such as TNFα, are known to induce the phosphorylation of RelA at multiple sites [77], [78], [79]. Using antibodies that specifically recognize serine 276 and serine 536 phosphorylated RelA, we were able to confirm that RelA is indeed phosphorylated at both of these sites in Tat-treated BV-2 cells, but that ibudilast does not affect this Tat-induced phosphorylation of RelA (Figure 6E). Although the precise mechanism of ibudilast's action on NF-κB transcriptional activity remains unknown, we concluded that ibudilast may block NF-κB activity in a manner that is independent of nuclear translocation and phosphorylation of RelA.

## Discussion

We, and others, have previously demonstrated that TNFα produced from HIV-1 infected or activated macrophage and microglia in the CNS is a major player in HIV-1-induced neuroinflammation, that eventually leads to neuron damage and cognitive impairment [54], [80], [81], [82]. Both neuroinflammation and monocyte/macrophage infiltration into the CNS are critical factors in the development of HAND, despite effective control of HIV-1 levels with HAART. Antiretroviral drugs target and effectively control viral replication, but once proviral DNA has integrated into the host chromosome, the production of early viral proteins such as Tat, a known neurotoxin, is not affected by these antiretrovirals [83]. It is also important to consider the limited CNS penetration and neurotoxicities of many antiretroviral drugs themselves [84], [85]. For these reasons, mechanisms to inhibit macrophage and microglial activation and neuroinflammation deserve further investigation in the pursuit of adjunctive therapies designed to protect CNS cells from damage caused by HIV-1. A myriad of possible adjunctive treatment compounds have been tested *in vitro*. Compounds targeting cellular signaling pathways including glycogen synthase kinase-3 beta (GSK3β) and mixed lineage kinase-3 (MLK3), as well as compounds targeting pathological outcomes of HIV-1 infection in the CNS including excitotoxicity, oxidative stress, and inflammation showed initial promise *in vitro*, however, only a handful of these treatments have moved into clinical trials. To date, only memantine, an NMDAR blocker, has preliminarily shown the ability to protect patients from HIV-1-induced neurocognitive damage [27]. The persistence of HAND despite effective HAART treatment, together with the lack of effective available adjunctive therapies for HAND, highlight the need for studies investigating novel adjunctive therapies.

In addition to ibudilast, other PDE inhibitors have been considered as anti-inflammatory agents in the context of HIV-1 infection. For example, initial *in vitro* experiments with pentoxifylline, a non-selective PDE inhibitor, showed that this drug inhibited microglial cell activation and the production of pro-inflammatory cytokines [86]. Also, in peripheral blood mononuclear cells (PBMCs) from HIV-1 positive, pentoxifylline-treated patients, there was lower LPS-induced TNFα release as compared to cells from HIV-1 positive, non-treated patients [36]. However, it was subsequently shown that the maximum tolerated dose of pentoxifylline in patients did not produce plasma concentrations high enough to match concentrations necessary to achieve the anti-inflammatory effects seen *in vitro* [87]. Additionally rolipram, a specific inhibitor of PDE 4, has been shown to suppress cytokine production and to inhibit HIV-1 replication in T-cells [35]. The low plasma concentrations of pentoxifylline, together with the gastrointestinal side effects associated with the use of PDE inhibitors, has limited further investigation of several PDE inhibitors for use in the context of HIV-1 infection.

NF-κB signaling plays an important role in the production of TNFα. As such, it has been shown that pentoxifylline inhibits NF-κB as a mechanism for its inhibition of TNFα [31], [88], [89], [90]. Also, increased cAMP levels have been shown to inhibit NF-κB transcriptional activity [91]. Considering this, we chose to investigate the effect of ibudilast on NF-κB as a potential mechanism for its inhibition of TNFα. As predicted, we did see a significant inhibition of Tat-induced NF-κB activation by ibudilast. Interestingly, we did not see a reversal of Tat-mediated IκBα degradation with ibudilast pre-treatment. This lack of IκBα stabilization by ibudilast suggests that the NF-κB inhibition by ibudilast occurs after RelA nuclear translocation, which is mediated by IκBα degradation. The transcriptional activity of RelA is partially controlled by multiple post-translational modifications, including phosphorylation, ubiquitination, and acetylation [76]. However, we did not see a reduction in Tat-induced RelA phosphorylation with ibudilast pre-treatment. Other possibilities for NF-κB inhibition in the nucleus include additional post-translational modifications, such as acetylation or ubiquitination, dimer exchange, or interactions with nuclear proteins such as the transcriptional coactivator and acetyltransferase, p300. Given the complexity of the regulation of NF-κB activity, it is not surprising that ibudilast's inhibition of NF-κB signaling appears to involve a post-translational modification of NF-κB, following normal mobilization of these molecules into the nucleus. Ibudilast could also potentially be inhibiting the DNA binding activity of NF-κB, as has been reported with the type 3 PDE inhibitor, cilostazol [52]. The precise mechanism of ibudilast's action on NF-κB transcriptional activity is currently under investigation in our laboratory.

In addition, there are hundreds of NF-κB inhibitors, all of which have greatly contributed to NF-κB research, but have limited clinical applications because of considerable toxicity to healthy cells, as NF-κB signaling is intricately involved in multiple cellular processes [92]. Considering the multiple functions of NF-κB signaling, specifically in the CNS [93], drugs such as ibudilast, with only partial inhibition of NF-κB in the context of HIV-1 in the CNS, could be beneficial.

Based on ibudilast's history of use in patients, this drug deserves further study as an anti-inflammatory agent in the context of HIV-1-induced neuroinflammation. Recent studies have shown that combinations of PDE inhibitors can synergistically suppress TNFα production by microglia at much lower concentrations than any single PDE inhibitor alone [32], [42]. This could circumvent the problem of low patient plasma concentrations encountered with pentoxifylline treatment. It is also important to emphasize the tolerability of ibudilast in patients, as only mild gastrointestinal side effects, such as nausea and diarrhea, have been reported [28], [29]. Indeed, ibudilast has been used for decades to treat bronchial asthma in Japan, and is being tested in clinical trials as a treatment for MS, opioid withdrawal, and neuropathic pain. Additionally, ibudilast has been shown to effectively cross the blood brain barrier and has been measured in rat brain tissue at concentrations close to plasma levels [30].

Considering ibudilast's potential ablity to inhibit or resolve CNS inflammation, there could be additional benefits such as limiting viral spread by reducing the inflammation-induced influx of monocytes into the CNS. In addition, TNFα and other related cytokines have wide-ranging neuromodulatory and even neuroprotective functions [94]. As such, ibudilast's partial inhibition of Tat-induced TNFα production could be particularly useful when considering this drug as an adjunctive therapy for HAND. Complete inhibition of TNFα production is undesirable, as this could negatively impact TNFα's ability to perform efficient host defense. In this respect, it has been shown that TNFα blockade can cause reactivation of tuberculosis (TB) in animal models [95], and it has also been suggested that inhibition of TNFα in patients with chronic hepatitis B infection (HBV) could worsen the disease, since TNFα is involved in viral clearance [95]. Furthermore, inhibition of TNFα in the context of other neuroinflammatory disorders such as MS and Alzheimer's disease (AD) has very recently been shown to be associated with progressive multifocal leukoencephalopathy (PML) and demyelination [96]. Taken together, these considerations again emphasize that ibudilast's partial inhibition of TNFα could be beneficial, rather than detrimental. In summary, our findings shed light on the mechanism of ibudilast's inhibition of Tat-induced TNFα production in microglial cells and may implicate ibudilast as a potential adjunctive therapy for the management of HAND.
